# Body Actions Change the Appearance of Facial Expressions

**DOI:** 10.1371/journal.pone.0108211

**Published:** 2014-09-24

**Authors:** Carlo Fantoni, Walter Gerbino

**Affiliations:** 1 Department of Life Sciences, Psychology Unit “Gaetano Kanizsa”, University of Trieste, Trieste, Italy; 2 Center for Neuroscience and Cognitive Systems@UniTn, Istituto Italiano di Tecnologia, Rovereto, Italy; University of Udine, Italy

## Abstract

Perception, cognition, and emotion do not operate along segregated pathways; rather, their adaptive interaction is supported by various sources of evidence. For instance, the aesthetic appraisal of powerful mood inducers like music can bias the facial expression of emotions towards mood congruency. In four experiments we showed similar mood-congruency effects elicited by the *comfort/discomfort* of body actions. Using a novel *Motor Action Mood Induction Procedure*, we let participants perform comfortable/uncomfortable visually-guided reaches and tested them in a facial emotion identification task. Through the alleged mediation of motor action induced mood, action comfort enhanced the quality of the participant’s global experience (a neutral face appeared happy and a slightly angry face neutral), while action discomfort made a neutral face appear angry and a slightly happy face neutral. Furthermore, uncomfortable (but not comfortable) reaching improved the sensitivity for the identification of emotional faces and reduced the identification time of facial expressions, as a possible effect of hyper-arousal from an unpleasant bodily experience.

## Introduction

Bodily interaction with everyday objects within the peripersonal space has powerful effects. It can specify social [1] and communicative intentions [2], the morphology of body schema [3], as well as object depth, object shape, and tactile sensitivity [4]. Furthermore, hand movement kinematics has been found to depend on subjective well-being [5], which suggests a link between action comfort and workplace productivity [6]. Here, we take a step further by examining the impact of *comfortable/uncomfortable* reaches on the perception of facial expression of emotions.

Even simple activities include complex sequences of goal-directed reaches involved in the correct picking up of objects. Though reaching is an essential and pervasive component of everyday actions, people are almost blind to motor effort involved in body motion, and largely ignore biodynamic components such as muscular strength and number of involved joints [7]. Though subtle, postural shifts associated with reaching can have a strong impact on perception and performance [8–10].

Central to our study are two apparently unrelated findings. First, it has been found that the subjective state of *comfort*/*discomfort* is related to the psychological mood state [11] and to the individual reaching mode, with perceived *discomfort* increasing as the number of body parts (muscles, joints) engaged in reaching increases [12]. In particular, it has been shown that beyond a critical distance (corresponding on average to the 90% of the maximal arm extension) reaching for an object becomes uncomfortable and negative mood states can arise [12]. Second, hyper-arousal from sensory stimulation (i.e., a higher level of arousal than in the normal awake state, induced by exposure to cold) can improve stereoacuity and contrast sensitivity [13], confirming that perceived emotions can potentiate the benefits of attention on sensory discrimination [14]. Similarly, hyper-arousal from action (relative to inaction) might improve the detection of subtle variations in the facial expression of emotions.

Two related questions are at the focus of our study. *Can the comfort/discomfort of previously performed reaches systematically bias the perception of facial expressions towards a positive (happiness) vs. negative (anger) valence? Can sensitivity to facial expressions be improved by the previous engagement in reaching?*

### Action, emotion, and facial expressions

A large body of research on object perception and representation refers to the processing of information within a given sensory modality and to its interaction with primitives, schemata, and other types of mental entities. For instance, current models of perceived facial expression of emotions are focused on visual information. One influential approach to the recognition of facial expression of emotions is based on the identification of sets of local and global image features matching with characteristics common to a given emotion category [15–17]. However, in ordinary conditions facial expressions of emotions are perceived while observers process a multitude of internal and external stimuli resulting from their active interactions with the environment. Consistent with the role classically attributed to action in the acquisition of object knowledge, the integration of information obtained during the perception-action cycle attracts a growing body of research [18]. Despite this growing interest, the effects of body actions on the perception of emotions from facial expression represent a largely unexplored territory.

Since bodily interaction with everyday objects within the peripersonal space has been shown to have powerful effects on perception, it is reasonable to expect that action modulates the perception of facial expressions, thus playing a pivotal role in human communication and cognition. [19] showed that the perception of spatial layout is influenced by the bodily state of the observer: hills may appear steeper and distances farther away to participants who are old, fatigued, or wearing a heavy backpack. [20] found that endorsing an expansive rather than contractive posture of the body can increase dishonest behavior. [4] found that depth perception can be modulated by arm representation induced by visuomotor adaptation in which participants execute reaching movements with the visual feedback of their reaching finger displaced farther in depth, as if they had a longer arm. Among others, such effects show that the brain integrates sensory signals from the body, quickly adapting to newly established body postures and using them as flexible anchors yielding the observer to a vivid impression of three-dimensionality and valence.

Furthermore, research investigating possible links between emotion and cognition suggests that emotional states can influence seemingly unrelated domains such as the hierarchical organization of vision [21]. Emotions are pervasive as well as contagious, and can be evoked while viewing or mimicking emotionally expressive faces [9,22]. The categorical perception and representation of emotionally expressive faces depend on mood [23], through mediating factors such as past experience [24], neutral faces [25], and music [26,27]. Such effects are consistent with the emotional response categorization theory [28], implying that humans are tuned to perceive things that are congruent with their emotional state. For instance, [29] found that music alters the perception of facial expression of emotions in a mood-congruent direction: the amount of rejection/sadness perceived in a neutral expression largely increased after participants were exposed to a sad music. Here, in a similar vein, we hypothesize that the temporary mood induced by comfort/discomfort associated with goal-directed actions can bias the perceived expression of emotional faces.

Our identification task required observers to classify a face as “happy” or “angry” after a novel *Motor Action Mood Induction Procedure* (MAMIP) based on performing a series of comfortable/uncomfortable goal-directed reaching actions. According to [12] we manipulated the comfort/discomfort of actions by varying the depth extent of goal-directed reaches. In every identification trial a face displayed an expression corresponding to a randomly selected position along a happy-to-angry morph continuum. If motor action is an effective mood inducer, identification should then be biased in a mood-congruent direction: *comfortable* actions should increase the probability that a neutral face appears to display a *positive* emotion (happiness), because of the positive mood induced by the positive action valence. Conversely, *uncomfortable* actions should increase the probability that a neutral face appears to display a *negative* emotion (anger), because of the negative mood induced by the negative action valence. The effectiveness of the MAMIP was thus tested using an objective measure based on facial emotion identification rather than a subjective measure based on self-description, to avoid well known problems related to the self-referential assessment of internal mood states; i.e., to “emotional self-awareness” [30,31]. In the present study the effect of action on mood was thus assessed through an implicit, rather than an explicit, measure based on the biased identification of facial emotions contingent on reaching comfort/discomfort. If mood affects performance then the direction of the bias should be similar to the one observed using other types of mood inducers (e.g., music), being positive when preceded by an inducer with positive valence (i.e., comfortable actions) vs. negative when preceded by an inducer with negative valence (i.e., uncomfortable actions).

Furthermore, it is known that performance is affected by arousal [32]. Increases in arousal have been shown to: (1) modulate the responsiveness of neurons in the early mice visual system [33,34]; (2) facilitate attentional mechanisms in tasks requiring sustained performance [35]; (3) improve stereo as well as contrast sensitivity in humans [13]. Luminance contrast on its own is known to provide important information for the recognition of facial expressions and identity [36]. A further direct link between the perception of facial expression of emotions and arousal has been recently revealed by studies on emotion perception abnormalities. [37] found that schizophrenic patients were more sensitive to angry facial expressions than control observers when processing facial expressions along the happy-to-angry morph continuum. In addition, the tendency of schizophrenic patients to assign emotional salience to neutral social stimuli has been found to correlate with their higher level of emotional arousal [38].

Based on such evidence we expected the precision in facial emotion identification to be higher when the task is preceded by reaching (relative to an inaction *baseline* condition without reaching) and, in addition, to be higher after uncomfortable reaching (requiring a high level of motor activation/*arousal*) is higher than after comfortable reaching (requiring a low level of motor activation/*arousal*). A similar response time asymmetry along the comfort-discomfort continuum was also expected in the facial emotion identification task, given that in general responses are faster at higher arousal levels [39,40]. With specific regards to the perception of facial expressions, personality types with higher arousal levels (e.g., individuals with high subclinical anxiety or with anxiety disorder) generally show a stronger anger superiority effect, with faster reaction times to threatening/angry faces [41] and an improved capacity to quickly process more threatening faces at once [42], compared to low trait-anxiety individuals.

## Experiments

### Rationale & Expectations

We tested our hypothesis that body action comfort/discomfort affects the perception of facial expression of emotions in four experiments. In Experiments 1 and 2 action comfort/discomfort was systematically manipulated during visually guided reaching movements under unrestrained body conditions, following the expectation that action valence during motor interaction induces a positive/negative mood that shifts perceived facial expressions in a congruent direction. We tested participants in a facial emotion identification task individually. In two successive blocks distinguished by reaches of opposite valence we measured the average Response Time (RT) to 6 levels of morphed expressions, as well as two indices of categorical perception along the happy-to-angry morphed face continuum: (i) the Point of Subjective Neutrality (PSN; i.e., the categorical boundary corresponding to the facial expression that led to equal probabilities of “happy” and “angry” responses) and the Just Noticeable Difference (JND, defined as half the morph interval between 16 and 84 per cent “angry” responses). In Experiment 1 participants performed 50 comfortable reaches (followed by the emotion identification block) and then 50 uncomfortable reaches (followed by another emotion identification block). The ordering of action type was reversed in Experiment 2, given that mood induction might have a long duration and the perception of changing facial expressions is affected by hysteresis [43].

The following hypotheses were considered:

- *H1)* In both experiments individual PSNs should be shifted in the direction opposite to action valence (for instance, after an uncomfortable action the PSN should correspond to a morphed face containing more happiness than anger relative to the PSN obtained after the comfortable action).
- *H2)* As an effect of hysteresis PSNs should be globally shifted towards happiness in Experiment 2, relative to Experiment 1, given that in Experiment 2 initial reaching acts were uncomfortable, possibly inducing a negative mood that biased the whole session, making slightly happy faces look neutral.
- *H3*) In both experiments we expected JNDs and RTs to be smaller after uncomfortable than comfortable reaches.
- Facial expressions of happiness and anger are known to have different hedonic impact [44,45]. A 2D morphing procedure like the one we used generates an image resulting from the linear interpolation of image features. Therefore, a 50 per cent morph (in which a fully happy expression and a fully angry expression of the same person are present in equal proportions) may not necessarily correspond to a facial expression experienced as neutral. One major aim of Experiment 3 was thus to identify the *baseline* values of PSN and JND by measuring accuracy and precision in the same facial emotion identification task utilized in Experiments 1 and 2, but in the absence of previously performed actions. Hence, the following hypotheses were included:
- *H4*) If goal-directed reaches have an arousing effect on performance, then the average JNDs obtained in Experiments 1 and 2 should be smaller than the *baseline* JND in Experiment 3.
- *H5)* If comfortable reaches empower our sense of motor skillfulness, thus contributing to the establishment of a more positive mood than the neutral mood experienced in the absence of action (Experiment 3 - *baseline* condition), then average PSNs after comfortable reaches in Experiment 1 should be shifted toward anger relative to the *baseline* PSN in Experiment 3. This hypothesis is based on the general idea that, relative to inaction, action is rewarding, if executed within the comfort range. Vice versa, PSNs after uncomfortable reaches in Experiment 2 should be shifted toward happiness relative to the *baseline* PSN in Experiment 3, since reaching outside the natural grasping range would induce a negative mood, as a direct product of discomfort or as an effect of experiential avoidance [46]. It should be stressed that the expectation of a positive effect of comfortable reaches (relative to the baseline measured in Experiment 3) critically follows from the idea that engagement in comfortable actions is more pleasant than the comfort associated to inaction.

Finally, Experiment 4 was run to validate our happy-to-angry morph continuum allowing us to extract another group *baseline* PSN using a different task and a different experimental setting: a large group of participants were asked to position every emotional face belonging to the morph set used in Experiments 1–3 on a 1–17 graphic rating scale (from happy to angry in *version A* and vice versa in *version B*).

#### Participants

Two groups of undergraduates (total number = 119) of the University of Trieste participated in the experiments. All had normal or corrected-to-normal vision and were naïve to the purpose of the experiment. Students in the first group (n = 30; women = 21, median age = 22, all right handed) were randomly assigned to Experiments 1–3 (Experiments 1 and 2, 9 participants each; Experiment 3, 12 participants) and received class credit for participation. The data of Experiment 4 were gathered in two classroom meetings with 19 (version A) and 70 (version B) psychology students (women = 64; median age = 20), who took part in a 90-min collective session.

The study was approved by the Research Ethics Committee of the University of Trieste (approval number 52) in compliance with national legislation, the Ethical Code of the Italian Association of Psychology, and the Code of Ethical Principles for Medical Research Involving Human Subjects of the World Medical Association (Declaration of Helsinki). Participants in Experiments 1–3 provided their written informed consent prior to inclusion in the study. Participants in Experiment 4 provided their oral informed consent before a data collection session included in lecture hours of an “introduction to perception” course. The request of oral consent formulated by the instructor (co-author WG) made explicit that people not willing to participate in the session should simply not accept the response sheet, without any consequence on the evaluation of their course attendance. The instructor specified that the required oral consent was a confirmation of the general agreement (included in the information about psychology undergraduate courses) that lectures would include classroom demonstrations and participations to short experiments, as an important part of activities directed to the fulfilment of standard learning outcomes. In Experiment 4 data were collected in a group session. Written consent (implying identification of every respondent) was redundant. Age and gender were the only elements of personal information included in the response sheet, reinforcing the emphasis on the anonymous treatment of data which was part of group instructions at the beginning of session. All students present in the classrooms accepted the response sheet and therefore behave as active participants in the data collection sessions of Experiment 4. Response sheets were filed as raw documents. The Ethics Committee of the University of Trieste approved the participation of regularly enrolled students to data collection sessions connected to this specific study. The Ethics Committee of the University of Trieste thus approved both the written informed consent used for Experiments 1–3 and the oral informed consent used for Experiment 4. Dataset is available as (Data S1).

#### Apparatus & Stimuli

**Figure 1:**
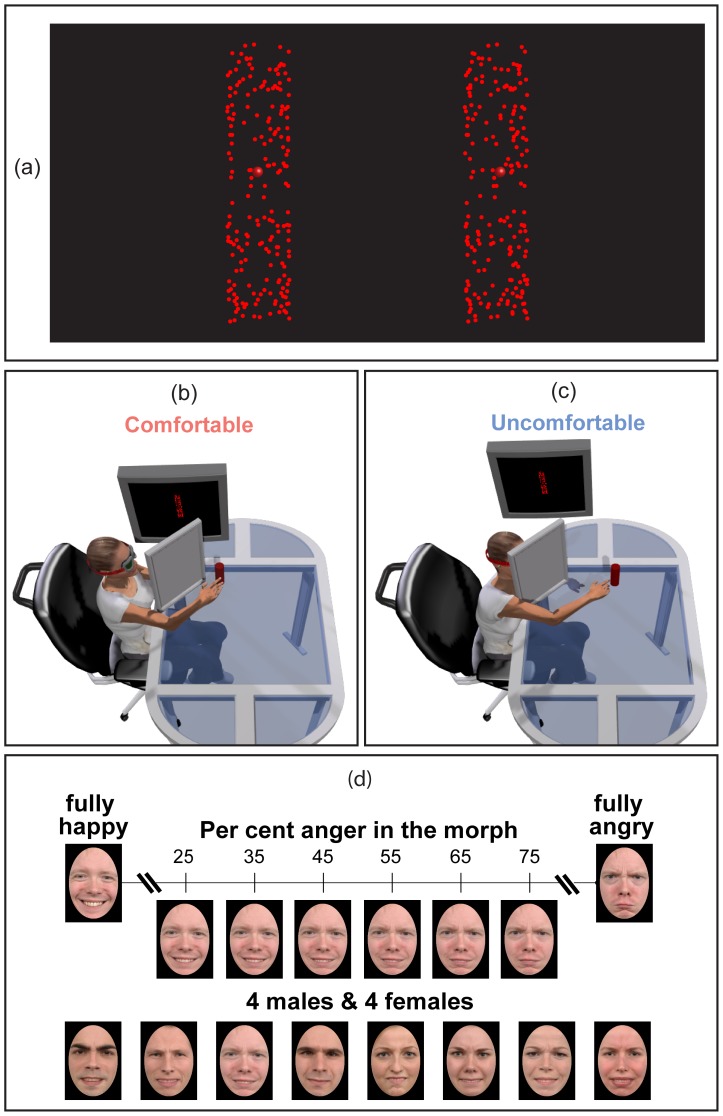
Random dot rod, action settings and facial stimulus set. A stereogram representing a frontal view of the random dot rod used in the our reaching blocks together with the red sphere used to provide a visual feedback of the index finger (cross-fuse) is shown in (a). A sketch of action settings used in comfortable (b) and uncomfortable (c) reaching blocks. The facial stimulus set is illustrated in (d): the top row shows the 6 faces of the happy-angry continuum (including percentages of extreme anger in the 25–75 per cent range, and complementary percentages of extreme happiness) and the fully happy (left) and fully angry (right) expressions used to generate the morph continuum, belonging to the fourth character of the bottom row; the bottom row shows the 8 characters selected from the Radboud database, displaying the “neutral” expression obtained by morphing the fully happy and fully angry expressions in equal percentages (50 per cent each).

In Experiments 1–3 participants were seated in a dark laboratory in front of a high-quality, front-silvered 40×30 cm mirror, slanted at 45° relative to the participant’s sagittal body mid-line and reflecting images displayed on a Sony Trinitron Color Graphic Display GDM-F520 CRT monitor (19″; 1024×768 pixels; 85 Hz refresh rate), placed at the left of the mirror (Figure 1b, c). For consistent vergence and accommodative information, the position of the monitor, attached to a linear positioning stage (Velmex Inc., Bloomfield, NY, USA), was adjusted on a trial-by-trial basis to equal the distance from the participant’s eyes to the virtual/real object that should be reached during the reaching block. To generate 3D visual displays we used a frame interlacing technique in conjunction with liquid crystal FE-1 goggles (Cambridge Research Systems, Cambridge, UK) synchronized with the monitor's frame rate. Head and index movements were acquired on-line with sub-millimeter resolution by using an Optotrak Certus motion capture system with two position sensors (Northern Digital Inc., Waterloo, Ontario, Canada). Head movements updated the participant’s viewpoint to present the correct geometrical projection of the stimulus in real time. The position of the index tip was calculated during the system calibration phase with respect to three infrared-emitting diodes attached on the distal phalanx. A custom C++ program was used for stimulus presentation as well as for the recording of response types (left/right keys of the computer keyboard) and RTs.

High-contrast random-dot visual stimuli were rendered in stereo simulating one vertically oriented rod with a dot density of 30 per cent and its back-surface visible (Figure 1a). The rod radius was 7.5 mm and the height 65 mm. The simulated egocentric depth of the rod axis along the line of sight was randomly chosen in the 0.65–0.75 range (Figure 1b), in the Comfortable block, and in the 0.90–1.00 range, in the Uncomfortable block (Figure 1c), relative to the arm length of each participant. The position of a physical rod (equal in shape to the virtual one) placed behind the mirror (completely occluded from the participant) was attached to a linear positioning stage (Velmex Inc., Bloomfield, NY, USA), adjusted on a trial-by-trial basis, so to align it perfectly with the virtual stimulus.

For our facial stimulus set (Figure 1d), we selected 8 characters (four Caucasian males and four Caucasian females) from the Radboud University Nijmegen set [47]. The colored photographs displayed facial expressions of two basic emotions, happiness and anger, all producing a high agreement of their intended expressions in the validation study. A happy-to-angry continuum was generated for each of the 8 characters, morphing the fully happy face and the fully angry face in variable proportions, in 5 per cent steps, using MATLAB software adapted from open source programs. Given two facial images and about 75 key points, the software generates a synthetic image that contains a specified mixture of the original faces, using a sophisticated morphing algorithm that implements the principles described by [48]. As in [49], we identified corresponding points in the two faces, with more points around areas of greater change with increasing emotional intensity (pupils, eyelids, eyebrows, and lips). For every character 6 morph intensities were selected along the happy-to-angry continuum, from 25 per cent angry ( = 75 per cent happiness) to 75 per cent angry ( = 25 per cent happy). All images were aligned for facial landmarks and masked by an oval vignette hiding hair and ears, presented on a black surround. The vignette was centered on the screen and had a size of 6.5×9.4 cm, corresponding to 7.5°×10.7° at the average viewing distance of 50 cm. Facial images used in each experimental trial were randomly extracted from this set of 48 stimuli (8 characters×6 facial expressions).

In Experiment 4 the same stimulus set was presented in a predefined pseudo-random order using PowerPoint through a high-resolution MARCA video projector connected to the graphic output of MAC-PRO (3D graphic accelerator). Participants were comfortably seated in a dimly lit classroom while facing the projection screen at the average distance of 12.25 m away. The average visual angle subtended by classroom displays was similar to the visual angle in Experiments 1–3, given that they were 35 times larger than the stimuli displayed on the lab CRT and the participant’s distance from the projection screen was about 35 times the one in the lab. Every participant was provided with a response form containing 48 numbered line segments, each with 17 equally spaced ticks (two extremes and central ticks marked in bold). Above the two extreme ticks two verbal labels were displayed: “happy” (left) and “angry” (right) for *version A,* and vice versa for *version B.* This manipulation was intended to control for possible effects of the spatial orientation of the rating scale.

#### Procedure

Reaching blocks (Experiments 1 and 2): The participant started a right hand movement from a fixed, out of view, position shifted relative to the body midline by about 25 cm from the sagittal plane and 15 cm from the coronal plane. The tip of his/her index finger, marked by a virtual red sphere (Figure 1a), was constantly visible from the moment the finger entered in the participant’s visual field. The task was to reach and touch the simulated random dot rod (Figure 1a) positioned along the line of sight (Figure 1b, c). Each successful reach was accompanied by haptic feedback (Figure 1b, c, red floating rod) and followed by acoustic feedback. Each block lasted 50 reaches, with the depth extent of each reach randomly selected in a range below (0.65–0.75 of arm length, Comfortable block) or above (0.90–1.00 of arm length, Uncomfortable block) the individual preferred critical boundary for one degree of freedom visually guided reaching [12], corresponding to the distance beyond which actors should introduce additional degrees of freedom to reach an object, with respect to those associated only to arm movements (Figure 1b, c).

**Figure 2:**
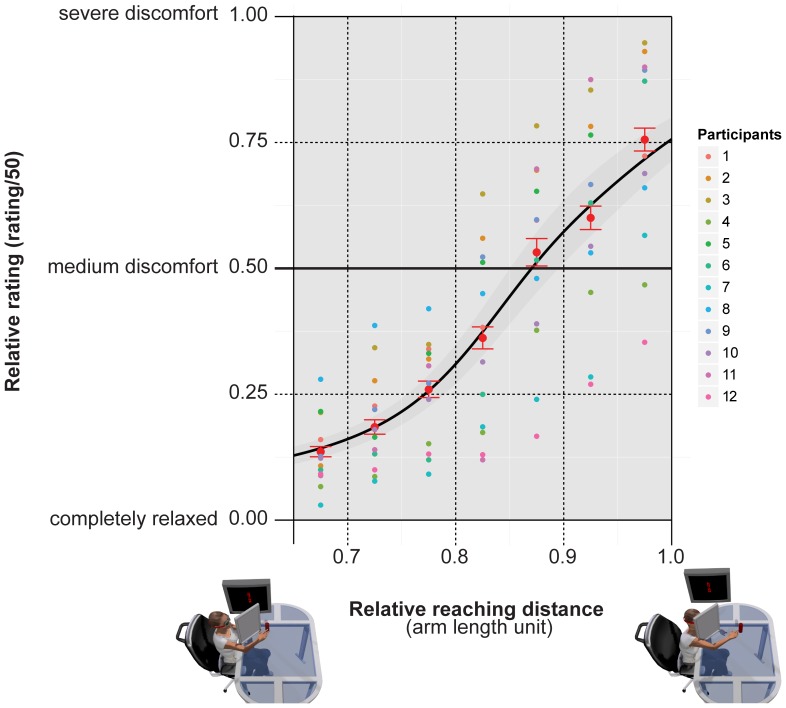
Subjective estimate of action discomfort increases with reaching distance. Average relative rating of action discomfort as a function of reaching distance (measured relative to individual arm length) collected in the preliminary experiment. Small dots represent individual color-coded average ratings for 7 equal intervals of relative reaching distance. The larger red dots represent the global average ratings ± SEM. The black line is the generalized linear model regression curve and the shaded region represents ± standard error of the regression.

The range of depths used for comfortable vs. uncomfortable actions were established empirically on the basis of the results of a preliminary experiment, in which 12 randomly selected students (6 women; median age = 23) of the University of Trieste were asked to perform 50 reaches toward the same random-dot cylinder used in Experiments 1 and 2, whose depth was randomly varied across trials in the entire 0.65–1.00 range of arm length (the same experimental setting of Experiments 1 and 2 was used). After each reach the participant was asked to rate the discomfort of the performed action on a 0–50 discomfort scale adapted from [50] pain scale (0 = reach felt completely natural; 25 = reach felt slightly unnatural as causing a moderate discomfort; 50 = reach felt completely unnatural as causing a severe discomfort). Figure 2 illustrates the average ratio between the rating and the maximum value of the scale (over 7 equal intervals of relative reaching distance) together with the best fitting sigmoid function whose parameters were extracted after modelling the whole set of individual responses using a generalized linear model based on a *Cauchy link* function with a variable slope and intercept for every participant.

Two main results are: (1) the entire range of depths used to manipulate the reaching comfort/discomfort (0.65–1.00 of arm length) produced a sizable effect on the subjective estimate of action discomfort as monotonically increasing with reaching distance for all tested participants (r^2^ = 0.86, slope = −8.53±1.15, intercept = 9.73±1.31, *df* = 554, *z* = 7.37, *p* = 0.0001); (2) the average distance (0.88±0.020) at which the cumulative function crosses the 0.5 response level was close to the preferred critical boundary for one degree of freedom visually guided reaching found by [12]. These preliminary results were in agreement with previous results showing that during reaching the lower is the amount of compensatory body movements not regarding the arm (such as shoulder or trunk) the larger is action *comfort* [51,^52^,12]. According to such results a person is in a state of postural comfort if there is not, and likely will not arise, a (possibly unaware) desire or need for compensatory motions of other body parts [7]. Furthermore, the results demonstrated that in our setup visually guided reaches were felt as comfortable in the 0.65–0.75 depth range and uncomfortable in the 0.90–1.00 depth range, thus setting the optimal conditions for the occurrence of opposite biases in the perception of facial expressions.

The procedure included: a session in which the participant’s arm length at rest (i.e., the effective maximum reach) was carefully measured following a procedure similar to the one used by [12] (see Appendix 1A in [12]), instructions, a training with 15 reaches randomly extracted across the entire depth range used in the experiment (0.65–1.00 of arm length), and the experimental session.

*Facial emotion identification task* (Experiments 1–3): In Experiments 1 and 2 the participant performed the required reaches and then the facial emotion identification task lasting 48 trials (approximately 10 minutes). In Experiment 3 the participant performed only the 48-trial facial emotion identification task, not preceded by reaching actions. Compared to Experiment 3, the facial emotion identification task in Experiments 1 and 2 thus involved more physical constraints (that might slow down responses): the participant should identify facial expressions right after the MAMIP, when his/her movements were still limited by infra-red markers, and his/her left hand and fingers should be positioned on the response pad by the experimenter. The 48 experimental displays resulted from the combination of 8 characters (4 actors and 4 actresses)×6 morph levels (from 25 to 75 per cent anger). The psychophysical method of constant stimuli was used in order to measure, for every participant, the PSN and JND for each of the 8 morph continua. Each facial emotion identification trial included the following: (1) a 30-pixel-wide green circle was displayed at the center of the screen for about 300 ms; (2) the face stimulus was displayed for 500 ms; (3) a blank screen followed (if the response were provided during the face presentation the blank screen lasted 200 ms); (4) until the participant pressed one of the two response keys with his/her left hand (left key for “happy” vs. right key for “angry”); (5) the next trial followed. The left hand was used for responses to the identification task given that in Experiments 1 and 2 the right hand, wearing markers, was used for the reaching task.

The experiments were run in a dark room allowing for dark adaptation. The participant was seated 50 cm away from the screen reflected in the mirror. The procedure included instructions, a training session in which the stimuli for the facial emotion identification task were the fully happy and fully angry faces of the 8 characters, presented twice in random order, and the experimental session.

*Rating scale task* (Experiment 4): The procedure was the same as in Experiment 3, except that participants were instructed to perform a different task on emotional face stimuli. Specifically, participants were carefully instructed to rate the amount of happiness/anger of each emotional face by crossing out the tick that marked the position along the happy-to-angry continuum corresponding to the displayed face.

## Results and Discussion

### Statistical analysis

In Experiments 1–3, indices of individual facial emotion identification performance were calculated by fitting a psychometric curve to individual data; i.e., to the percentage of “angry” responses as a function of the percentage of full anger in the 6 sets of morphed faces (each including 4 males and 4 females). Curve fitting followed the procedure indicated by [53]. We modelled the whole set of binary responses using a generalized linear model with a *probit link* function with variable slope (β_1_) and intercept (β_0_) for every combination of participant, reaching block, and experiment. Then, we reparametrized each individual Gaussian function fit in term of its mean (−β_0_/β_1_) and standard deviation (1/β_1_). The mean defined the PSN along the happy-to-angry continuum, corresponding to equal probabilities of obtaining “happy” and “angry” responses (i.e., to maximum uncertainty). The standard deviation defined the JND.

**Figure 3:**
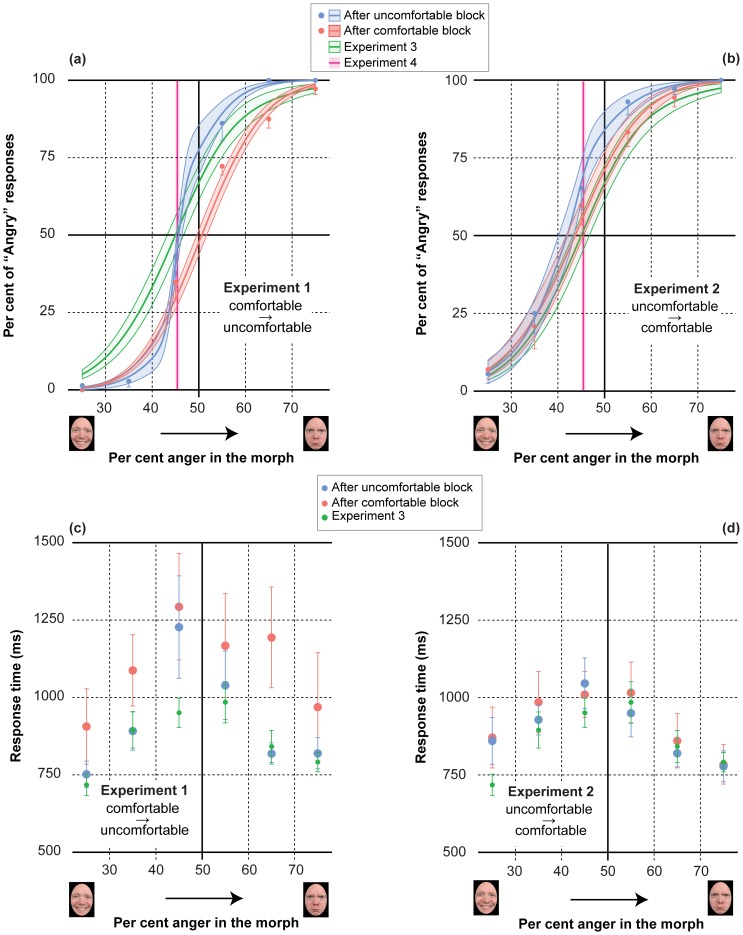
Distributions of percentages of “angry” responses and RTs. The 4 panels depict the average percentages of “angry” responses (a, b panels) and RTs (c, d panels) [± SEM] as a function of per cent anger, after the comfortable/uncomfortable (red/blue symbols, respectively) reaching blocks and in the absence of action (green symbols). Red and blue curves in a, b panels are the best average cumulative Gaussian fits of response percentages, with shaded bands indicating ± standard error of regression. Green curves represent the average distributions, ± SEM, obtained in Experiment 3. The pink line represents the average PSN, ± SEM, obtained in Experiment 4. Data in the left panels (a, c) refer to Experiment 1 (comfortable-uncomfortable order); data in the right panels (b, d) refer to Experiment 2 (opposite order).

Panels a, b in Figure 3 illustrate the average percentage of “angry” responses together with the best fitting cumulative Gaussian as a function of per cent anger for comfortable (red) vs. uncomfortable (blue) actions, for the two orderings of reaching blocks: comfortable-uncomfortable (panel a) vs. uncomfortable-comfortable (panel b). As an index of identification precision we used the JND, corresponding to the standard deviation of the best fitting Gaussian model (smaller JND indicating higher identification precision). To provide an additional converging measure of the possible effect of action-induced mood on facial identification performance we also analyzed individual RTs (taking as valid RTs those between 200 and 4000 ms, which led to the removal of 44 out of 2592 values collected over Experiments 1–3) averaged for each of the 6 morph levels (c and d panels in Figure 3, for Experiments 1 and 2, respectively).

**Figure 4:**
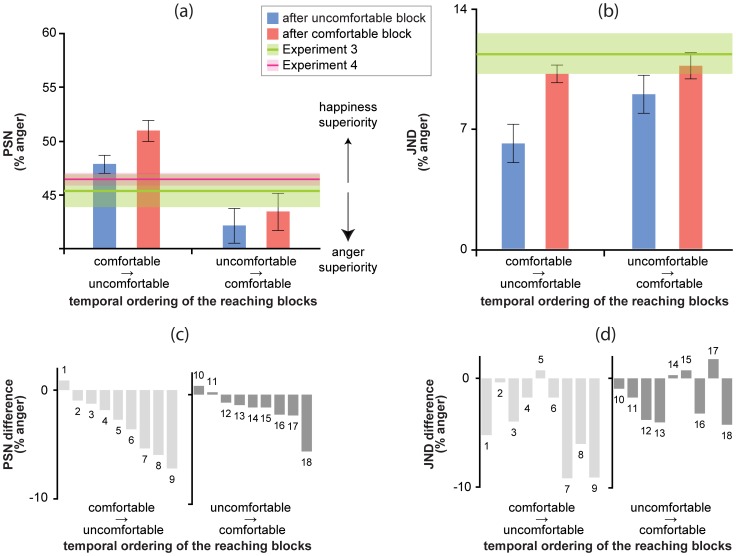
Action comfort/discomfort biases the perception of facial emotions. Average PSNs (a) and JNDs (b), ± SEM, for the comfortable (red) and uncomfortable (blue) reaching blocks in Experiments 1 (comfortable → uncomfortable) and 2 (uncomfortable → comfortable) as coded along the x-axis. Horizontal green and violet lines represent the baseline scores, ± SEM, obtained in Experiments 3 and 4. In (a) these scores are the reference for evaluating the biasing effects of action comfort/discomfort, with PSNs larger than the baseline indicating an overall happiness superiority, and PSNs smaller than the baseline indicating an anger superiority. In (b) values below the green line indicate a precision improvement induced by the reaching block. (c) Individual PSN difference between uncomfortable and comfortable reaching sessions in Experiments 1 (light grey) and 2 (dark grey). A negative value represents an increased likelihood of perceiving a facial expression as being angry after the uncomfortable block. (d) Individual JND difference between uncomfortable and comfortable reaching sessions in Experiments 1 (light grey) and 2 (dark grey). A negative value represents a stronger improvement in facial expression sensitivity after the uncomfortable (rather than comfortable) block.

Figure 4 shows the average PSNs and JNDs for the two reaching blocks in Experiments 1 (comfortable block first) and 2 (uncomfortable block first), relative to baseline values obtained in Experiments 3 and 4. We analyzed PSNs and JNDs using a linear mixed-effect (*lme*) model with participants as random effects, and reaching block (comfortable vs. uncomfortable) and Experiment (1 vs. 2) as fixed effects [54,55]. A similar *lme* analysis was applied to RTs, using the per cent anger in morph as a fixed factor to manage the intrinsic nonlinearity between RT and morph intensity. Data of Experiment 4 have been first converted into a –50 (fully happy) to 50 (fully angry) scale and then analyzed using a *lme* model with both participant and actor as random effects and per cent anger in our stimulus set and the version of the rating scale (A vs. B) as fixed effects. We used type 3 like two tailed *p*-values adjusting for the *F*-tests the denominator degrees-of-freedom with the Kenward-Rogers approximation implemented in KRmodcomp's function, R Package pbkrtest [56,57]. Among the indices that have been proposed as reliable measures of the predictive power and of the goodness of fit for *lme* models (e.g., [58]) we selected the concordance correlation coefficient, *r_c_*, providing a measure of the degree of agreement between the observed values and the predicted values, in the –1 to 1 range [59]. Post-hoc tests were performed using two tailed *t*-tests and Cohen's *d* as a measure of significant effect size.

### Biasing the perception of facial emotion through action comfort/discomfort

Average PSNs shown in Figure 4a were in strong agreement with *H1*: the PSN was indeed biased in opposite directions after comfortable (towards anger) vs. uncomfortable (towards happiness) reaching blocks in Experiments 1 and 2. In Experiment 1, the likelihood of interpreting a facial expression as angry increased by about 130 per cent (odds ratio) after participants were adapted to uncomfortable reaching acts, with average PSNs measuring 50.9±0.97 per cent anger and 47.7±0.83 per cent anger (*F*_1,8_ = 12.31, *p* = 0.007), after comfortable and uncomfortable reaching blocks, respectively. The effect was strikingly similar in Experiment 2, where the odds of an “angry” response after the uncomfortable reaching block outperformed those after the comfortable reaching block by 116 per cent, with average PSNs measuring 43.4±1.94 per cent anger and 42.0±1.83 per cent anger (*F*_1,8_ = 5.5, *p* = 0.04), after comfortable and uncomfortable reaching blocks, respectively. Consistently with the effectiveness of our MAMIP and with perceptual hysteresis (*H2*), we found a lower PSN in the uncomfortable-comfortable reaching condition (Experiment 2, 42.71 per cent anger) than in the comfortable-uncomfortable reaching condition (Experiment 1, 49.31 per cent anger).

The above described effects of motor action mood induction on PSN were predicted by the *lme* model with Experiment as a fixed effect revealing significant main effects for Reaching (F_1,16_ = 17.62, *p* = 0.0007) and Experiment (F_1,16_ = 10.58, *p* = 0.005), but not their interaction (F_1,16_ = 2.95, *p* = 0.104). Only 50 reaching acts distributed over 10 min, with a slightly different depth extent (average depth difference between comfortable and uncomfortable reaches = 17.74 cm ±0.19) produced dramatic changes in the perception of facial expressions.

However, a baseline *lme* model revealed a systematic bias in identification performance towards anger in Experiments 1 and 2, with an estimated PSN (averaged across experiments) of 46.02±1.26 per cent anger (*t* = 36.26). Given such a bias, we wondered whether it was due to our MAMIP or whether it was in line with a well known phenomenon in the emotion perception literature: angry faces “pop out” of crowds [60]. To address this question we contrasted the average PSN from Experiments 1 and 2 with that obtained in Experiment 3 (45.5±1.7 per cent anger), where a similar anger superiority effect was found even in the absence of previously performed reaches (Welch Two Sample *t* = 0.29, *df* = 17.51, *p* = 0.77). A similar result was also found in Experiment 4, where we used a different measurement method (rating scale task) and performed the experiment in the field (classroom), rather than in the laboratory. Average PSNs as extracted from an *lme* model with the per cent anger in our stimulus set as the only continuous predictor (*slope* = 1.21, *F*_1,3993_ = 7534, *p* = 0.000, *r_c_ = *0.84), revealed no effect of the ordering of the rating scale (*F*_1,87_ = 0.77, *p* = 0.38). A similar bias toward anger was observed when the response scale was reversed, and anger was presented on the left (version A: 46.19±0.67 per cent) or right of the scale (version B: 47.2±0.97 per cent). Again, the magnitude of the anger superiority effect revealed by Experiment 4 was about the same as the one obtained in Experiments 1 and 2 (PSN = 46.40±0.49 per cent, Welch Two Sample *t* = 0.56, *df* = 82.98, *p* = 0.57).

In summary, the present results reveal a symmetric bias in the perception of facial expressions, induced by comfortable/uncomfortable reaches. Consistently with *H5*, a sequence of comfortable reaches performed before the facial emotion identification task induced an increased likelihood of interpreting a facial expression as happy relative to the baseline. By contrast, uncomfortable reaches induced an increased likelihood of interpreting a facial expression as angry.

### Improving precision through action comfort/discomfort

To assess the impact of *hyper-arousal from reaching* on human ability to identify subtle facial expressions of emotion, we analyzed the JNDs and RTs in the absence of (Experiment 3) and immediately after the reaching blocks (Experiments 1 and 2). Three plausible patterns of results were considered:

1. Consistent with *H4*: INDs and RTs in Experiments 1 and 2 smaller than those in Experiment 3;
2. Consistent with *H3*: JNDs and RTs after the uncomfortable reaching block smaller than JNDs and RTs after the comfortable reaching block;
3. Inconsistent with both *H3* and *H4*: Neither JNDs nor RTs smaller after an uncomfortable reaching block (inducing hyper-arousal).

The first pattern of results would suggest that goal directed reaches can influence arousal, triggering an arousal-based improvement in emotional face processing, revealed by an increased sensitivity to facial expression differences (measured by the JND in the classification task), and by a reduction of the degree of uncertainty in emotion classification (measured by RTs). The second pattern of results would suggest that arousal can be modulated continuously by the nature of goal directed reaches, being it comfortable or uncomfortable. In contrast, the last pattern of result would suggest that there is likely limited benefit for arousal states from reaching actions. Average JNDs shown in panel b of Figure 4 are in good agreement with hypotheses *H3* and *H4*: participants’ sensitivity to subtle facial expression differences improved after both reaching blocks, but the improvement was larger after the uncomfortable, not comfortable, sequence of reaches. The distributions of average RTs depicted in panels c and d of Figure 3 provide converging evidence in support of hypothesis *H3*: participants indeed responded more quickly, thus showing an increased degree of certainty in performing the emotion identification task, after the uncomfortable sequence of reaches than after the comfortable.

In Experiment 1, the JND after being adapted to uncomfortable reaches was about half the one after comfortable reaches (from 10.22±0.5 per cent anger to 6.15±1.12 per cent anger; *F*_1,8_ = 11.41, *p* = 0.009). A similar although smaller effect was found in Experiment 2 in which the JND decreased by about 16 per cent after uncomfortable rather than comfortable reaches (from 10.7±0.76 per cent anger to 9.0±1.11 per cent anger; *F*_1,8_ = 5.1, *p* = 0.048).

In Experiment 1, RTs were similarly affected by both the mood induced by body action (*F*_1,88_ = 9.30, *p* = 0.003) and by the per cent anger in the morph (*F*_5,88_ = 5.08, *p* = 0.0004), with faster RTs after the uncomfortable (929±42 ms) rather than the comfortable (1103±63 ms) reaching block. RTs followed an inverted U-shaped function of per cent anger reaching a maximum (1273±115 ms) at 45 per cent anger, which is close to the average value of maximal response uncertainty. This was confirmed by post-hoc paired *t*-tests: RTs decreased by about 445 ms (paired *t* = −5.2, *df* = 17, *p* = 0.000, *d* = 1.12) as the per cent anger deviates from 45 per cent towards happiness, and by about 378 ms (paired *t* = −4.2, *df* = 17, *p* = 0.0005, *d* = 0.86) as the per cent anger deviates from 45 per cent towards anger. In Experiment 2, we found a similar, though not significant (*F*_1,88_ = 0.6, *p* = 0.50), tendency of uncomfortable reaching in reducing RTs (921±36 ms vs. 897±28 ms after comfortable vs. uncomfortable reaches), and a similarly strong modulation of RTs by the per cent anger in the morph (*F*_5,88_ = 5.98, *p* = 0.000).

The different effect sizes in Experiments 1 and 2 were likely due to the unbalanced temporal ordering of reaching blocks. In Experiment 2 our participants were more experienced with the experimental task after the comfortable rather than uncomfortable block, and vice versa in Experiment 1. The effects of action comfort and learning were thus in opposite directions in Experiment 2, thus reducing the performance difference induced by the two reaching blocks, and in the same direction in Experiment 1, thus enhancing the performance difference induced by the two reaching blocks.

We further demonstrated an arousal-based improvement in emotional face processing induced by reaching discomfort by the results of the *lme* model comparing the JNDs and RTs in Experiments 1 and 2. The model on JNDs revealed a significant main effect of Reaching (*F*_1,16_ = 16.27, *p* = 0.001); while neither the effect of Experiment (*F*_1,16_ = 2.40, *p* = 0.14) nor the Reaching × Experiment interaction (*F*_1,16_ = 2.86, *p* = 0.11) were significant. Similar results were obtained on RTs, in which Reaching (RT after comfortable = 1012±37 ms; RT after uncomfortable = 913±25 ms; *F*_1,176_ = 9.19, *p* = 0.003) and per cent anger in the morph (*F*_5,176_ = 9.05, *p* = 0.0000) were the only significant main effects; other effects were not statistically significant.

Consistent with the idea that arousal is mainly influenced by uncomfortable reaches, we found that the *baseline* JND obtained in Experiment 3 (11.14±1.46 per cent), in which performance was measured at the normal awake arousal state, was larger than the JNDs of the uncomfortable reaching condition averaged across Experiments 1 and 2 (7.59±0.84 per cent, Welch Two Sample *t* = −2.10, df = 18.2, *p* = 0.049), but not of the comfortable reaching condition (10.46±0.45 per cent, Welch Two Sample *t* = −0.44, df = 13.1, *p* = 0.66). Analogously, despite the larger number of physical constraints to which the observer was subjected in Experiments 1 and 2 relative to Experiment 3, which should determine an unbalance between conditions in favor of Experiment 3, RTs after uncomfortable reaches were identical to those observed in Experiment 3 (913±25 vs. 864±22; Welch Two Sample *t* = 1.47, *df* = 176.7, *p* = 0.14), while those after comfortable reaches (1012±37) were larger (Welch Two Sample *t* = 3.43, *df* = 166.2, *p* = 0.0007, *d* = 0.47).

In summary, we obtained three findings: (a) comfort/discomfort associated to goal-directed reaching biased the identification of facial emotions towards mood congruency; (b) discomfort (but not comfort) improved the precision of emotion identification; (c) discomfort speeded up the processing of facial expressions of emotion by reducing RTs and response uncertainty in our emotion identification task.

## Discussion

The present study demonstrates that *comfort/discomfort* of goal-directed reaching affects the perception of facial expression of emotions. Uncomfortable actions modified the perception of emotional expressions along the happy-to-angry continuum, making a neutral face appear angry and a slightly happy face neutral, and improving the identification of facial expressions. Comfortable reaching induced an opposite shift of the perceived midpoint of the happy-to-angry continuum, making a neutral face appear happy and a slightly angry face neutral, but without improving the identification of facial expressions.

Such biasing effects of action comfort/discomfort are challenging for the current approach to sensory integration, which is based on optimal cue integration [61–63] and on a view of the brain as a Bayesian inference system [64,65]. According to such an approach, the brain is continuously predicting the most likely interpretation of new visual inputs on the basis of expectations and beliefs about the environment, providing priors that are optimally combined with sensory evidence. But knowledge-based priors and sensory inputs are not enough as our results demonstrate that affective components cannot be ignored when considering the process of sensory integration.

Our results show that body feelings impact perception too, which is also consistent with recent findings on the effect of body posture on behavior [20] and the constructionist hypothesis by [66]. In particular, perceived affordances depend on body capabilities that are defined by the geometry (e.g., arm length) and biodynamics (e.g., muscular strength, joint mobility) of relevant parts of the actor's body. In the case of reaching, beyond a critical distance the arm is no longer sufficient; to reach farther, actors must activate other body segments, by either leaning forward or twisting their bodies to extend their shoulders towards the object. Above such a critical distance reaching becomes uncomfortable [12] and negative mood states arise [11], setting the stage for mood-congruency effects in emotion perception. On the other hand, the positive effect of comfortable reaches relative to the inaction condition measured in Experiment 3 can be interpreted as a by-product of the empowerment of motor skillfulness. Remarkably, our effect suggests that comfortable/uncomfortable actions can be conceived as a new powerful mood inducer. Hence, our Motor Action Mood Induction Procedure, MAMIP, should be added to the list including the Musical Mood Induction Technique, MMIT [67], the Velten Mood Induction Procedure, VMIP [68], and the self-referential mood induction [69], to name only a few procedures used in controlled settings.

Similar mood-congruency effects have been previously shown to occur using other mood-inducing procedures [70]. Our MAMIP is apparently new as an experimental setting (despite being implicit in all uses of relaxation as a route to well-being) and possibly more basic than others (given that listening to music – a powerful mood-inducer – evokes motor actions). Note also that music, verbal descriptions, and personal memories may be explicitly related to social perception; while the type of motor actions (i.e., reaches with slightly different depth extents) used as mood inducers in our study have no direct link with social perception, but still produce strong effect on emotion identification: reaching comfort/discomfort, as defined by the amount of compensatory body movements not regarding the arm, affects the individual mood state, which in turn influences the perceptual processing of facial expression.

There are two ways of looking at the mood-congruency effects we demonstrated in our study. Action-induced mood might affect only post-perceptual processing by modifying the response criterion and decision thresholds or mood might affect valence through a top-down modulation of visual processing in which perception is directly influenced by the observer’s psychological state [71]. Although our study is compatible with both hypotheses, we suggest that the second is more intriguing as it sheds light on new links between perception and action. Classic research focused on the role of vision for the control of fundamental motor action that humans perform with great dexterity, such as reaching and grasping [72]. On the other hand, important work has been conducted on visuomotor adaptation showing how hand proprioception might alter basic perceived object properties, such as shape, position, and size [4]. Our study provides the first evidence that expressive qualities of the social environment can be altered by subjective feelings associated to motor actions.

Our results are consistent with the pioneering idea that muscular and somatic states might constitute hard representations used in high level cognition [73]. If the motor system is representational in nature then performing an *uncomfortable* action is likely to evoke facial expressions with negative valence, thus selectively tuning the perceiver towards face stimuli with an expression that is congruent with the one activated by the action itself.

However, given that no traditional explicit measures of subjective mood were collected in the present study (see [30] for a review), it is possible that action comfort/discomfort could have biased the perceived facial expressions without influencing mood. However, this seems unlikely, as the behavioural effects of our action-based induction were similar to those of other mood inducers (e.g., music). An interesting issue for further research is thus to clarify the mediator effects of variables such as mood, experiential avoidance, sense of reward, and sense of motor skillfulness.

Furthermore, the improvement of emotion identification performance induced by action comfort/discomfort suggests that one way action might affect the perceptual system is through arousal, which can prompt vision and attention enhancing detection capabilities. This finding is in line with the evidence that hyper-arousal from sensory stimulation can influence aspects of human visual perception [13,35]. One way in which arousal might have affected the performance in our task is by a modulation of attention which is known to be linked to emotion and in particular mood [74,75]. Mood was shown to affect attention through determining the focus of processing of visual stimuli [21] favoring a local processing strategy under negative mood state (i.e., uncomfortable block), vs. a global processing strategy under positive mood state (i.e., comfortable block). The improvement of performance in the uncomfortable relative to the comfortable block, revealed by our study, is thus in-line with recent findings showing that observers primed with local processing performed both significantly faster and more accurately on emotion recognition tasks than when they were primed with a global processing [76].

In summary, models of perception-action interaction should include emotion to predict, in particular, arousal-based changes of identification performance. In particular our results suggest a challenge in the interpretation of those numerous studies comparing perceptual based estimates vs. action based estimates of size [77]. For instance, the finding that estimated depth with the index-to-thumb span is larger when the observer is asked to actively reach and grasp for a target object rather than to indicate the depth of the object while holding their hand away from it [78], could be a by-product of an enhancement of stereo sensitivity caused by the increased arousal induced by visually guided reaches.

Our findings have practical implications for the interior design of houses and workplaces, and exemplify a causal effect of action on perception relevant for *emotional design* [79]. The mood induced by comfortable/uncomfortable actions on/with daily objects affects the valence and discriminability of the expressive features of external objects, including conspecifics. Consider workplaces where actions are constrained by the physical structure of the environment. Comfortable artefacts at an easy-to-reach distance would induce a positive mood, which in turn would enhance the global experience of pleasantness, as revealed by a bias in perceiving faces as pleasant (happy) rather than unpleasant (angry). Among other undesirable effects, body discomfort induced by bad interior design degrades our social environment.

## Supporting Information

Data S1 **Data from Experiments 1–4.** Two worksheets are included in the file: (1) RAW_DATA_EXP12&3, with the entire dataset of Experiments 1–3, and (2) RAW_DATA_EXP4, with the entire dataset of Experiment 4.
(XLS)
